# A study protocol for a pilot randomised trial of a structured education programme for the self-management of Type 2 diabetes for adults with intellectual disabilities

**DOI:** 10.1186/s13063-015-0644-y

**Published:** 2015-04-10

**Authors:** Laurence Taggart, Vivien Coates, Mike Clarke, Brendan Bunting, Melanie Davies, Marian Carey, Ruth Northway, Michael Brown, Maria Truesdale-Kennedy, Lorraine Martin-Stacey, Gillian Scott, Thanos Karatzias

**Affiliations:** Institute of Nursing and Health Research, Ulster University, Shore Road, Newtownabbey, Co Antrim BT37 0QB Northern Ireland; MRC Hub for Trials Methodology Research, Queen’s University Belfast, Malone Road, Belfast, BT7 1NN Northern Ireland; Institute of Psychology, Ulster University, Northland Road, Derry, BT48 7JL Northern Ireland; Leicester Diabetes Centre, University of Leicester, Gwendolen Road, Leicester LE5, England; University of South Wales, Glyntaff Campus, Cardiff, CF37 4BD Wales; Edinburgh Napier University, Sighthill Campus, Bankhead Avenue, Edinburgh, EH11 4DE Scotland; Northern Health and Social Care Trust, Ulster, Rathlea House, Mountfern Complex, 8A Rugby Ave, Coleraine, BT52 1JL Northern Ireland

**Keywords:** Intellectual disability, type 2 diabetes, self-management, structured education

## Abstract

**Background:**

The need for structured education programmes for type 2 diabetes is a high priority for many governments around the world. One such national education programme in the United Kingdom is the DESMOND Programme, which has been shown to be robust and effective for patients in general. However, these programmes are not generally targeted to people with intellectual disabilities (ID), and robust evidence on their effects for this population is lacking. We have adapted the DESMOND Programme for people with ID and type 2 diabetes to produce an amended programme known as DESMOND-ID.

This protocol is for a pilot trial to determine whether a large-scale randomised trial is feasible, to test if DESMOND-ID is more effective than usual care in adults with ID for self-management of their type 2 diabetes, in particular as a means to reduce glycated haemoglobin (Hb1Ac), improve psychological wellbeing and quality of life and promote a healthier lifestyle. This protocol describes the rationale, methods, proposed analysis plan and organisational and administrative details.

**Methods/Design:**

This trial is a two arm, individually randomised, pilot trial for adults with ID and type 2 diabetes, and their family and/or paid carers. It compares the DESMOND-ID programme with usual care. Approximately 36 adults with mild to moderate ID will be recruited from three countries in the United Kingdom. Family and/or paid carers may also participate in the study. Participants will be randomly assigned to one of two conditions using a secure computerised system with robust allocation concealment. A range of data will be collected from the adults with ID (biomedical, psychosocial and self-management strategies) and from their carers. Focus groups with all the participants will assess the acceptability of the intervention and the trial.

**Discussion:**

The lack of appropriate structured education programmes and educational materials for this population leads to secondary health conditions and may lead to premature deaths. There are significant benefits to be gained globally, if structured education programmes are adapted and shown to be successful for people with ID and other cognitive impairments.

**Trial registration:**

Registered with International Standard Randomised Controlled Trial (identifier: ISRCTN93185560) on 10 November 2014.

## Background

Type 2 diabetes (T2D) affects approximately one in twenty people across Europe and accounts for an uneven use of healthcare resources [15]. Traditional management of T2D is based on medication, diet and lifestyle modifications, and managed by the patient’s GP, practice nurse and/or diabetes specialist nurse (DNS), with three monthly visits to the health centre [6]. However, many patients find this management strategy difficult to implement and sustain [22]. The need for structured, rather than *ad hoc*, patient education programmes for diabetes using both a theoretical rationale and cognitive reframing has a high priority on the governmental healthcare agenda in the United Kingdom ([10,21]. One specific national programme, the Diabetes and Self-Management for Ongoing and Newly Diagnosed patients with T2D Programme (DESMOND), has been shown to be a robust and effective programme for those with T2D, illustrating improvements in weight loss, depression, smoking cessation, beliefs about diabetes and greater perceived responsibility [5,11,16,23,28].

There is growing evidence to show that as people with ID live longer, they are becoming more susceptible to a range of chronic physical health conditions, such as respiratory disease, cardiovascular disease, gastrointestinal and gall bladder cancers, and T2D [30,38]. Although limited information exists on the incidence of diabetes in people with ID, McVilly *et al*. [20] in a systematic review reported that people with ID are more likely to develop T2D compared to their non-ID peers: approximately 8% (range: 3% to 18%) rather than 4% in the non-disabled population [15]. This increased risk may result from the ID population being more likely to have poor nutrition, consume high fat foods, undertake less physical activity and lead more sedentary lifestyles, resulting in greater levels of obesity [13,34,38]). Lennox *et al*. [17] in Australia, Shireman *et al*. [26] in Canada and Taggart *et al*. [32] in Northern Ireland found that a considerable proportion of people with ID living in the community also had T2D, which was poorly managed. Further, people with ID have had few opportunities to engage in diabetes health education and screening [26,32,33].

Internationally, reports have highlighted the continued neglect of the health of people with ID, and that healthcare services consistently fail to work together and make reasonable adjustments to meet the health needs of this population [7]. With a national focus on health disparities and health promotion in the non-disabled population, it is important that health issues that have not been addressed are identified and acted upon for all vulnerable populations; further illustrating the ongoing health inequalities this population face [9].

The robust structured education programmes developed to improve the biomedical, psychosocial and self-management strategies for non-disabled people with type 1 and 2 diabetes have not been targeted to adults with ID [32,]. For example, Taggart, Brown & Karatzais (2014) [33] reported that there was a lack of appropriate information on diabetes and diet management for people with ID, and that little emotional support was being offered to adults with T2D who had ID. People with ID rarely accessed structured education programmes for diabetes that were offered to the non-disabled population [29]; further illustrating ongoing inequality and disadvantage [8]. These programmes have neither recognised nor addressed the specific challenges posed by this population’s cognitive deficits, communication difficulties, low levels of literacy skills and different learning styles. There is also a lack of adequate attention to the need to support family and paid carers [13].

A search for other research found no trials pertaining to diabetes education for people with ID in the World Health Organization’s International Clinical Trials Registry Platform [24]. Likewise, we have not identified any structured education programmes being delivered internationally that are specifically tailored to the needs of adults with ID and T2D, or that target the carers who support this population. In view of the burden and severity of diabetes, it is essential that adults with ID and their family and/or paid carers are afforded the opportunity of an evidence-informed education programme that meets the National Institute of Clinical Excellence Standards [21].

### Study objectives

With funding from Diabetes UK, Taggart and colleagues have adapted the DESMOND Programme so that it could be used for adults with ID in the United Kingdom. They developed DESMOND-ID through robust delivery and evaluation of the original DESMOND education programme over 18 months, using video-recordings, focus groups with service users and their carers, feedback from two DESMOND Programme educators and observations from three independent health professionals.

The key research question is: Can we design a feasible, large-scale randomised trial that will resolve uncertainty and determine whether DESMOND-ID is more effective than usual routine care in helping adults with ID to better self-manage their T2D to reduce their Hb1Ac? In order to answer this question, we will conduct a small pilot randomised trial comparing the use of the DESMOND-ID programme with usual routine care across three countries in the United Kingdom (Northern Ireland, Scotland and Wales). This pilot trial will include carefully selected secondary outcome measures (body mass index (BMI) waist circumference, blood pressure, lipid profiles, cardiovascular disease (CVD) risk score, smoking, alcohol use, exercise, perceptions and severity of beliefs, and quality of life), in addition to the primary outcome measure of Hb1Ac.

People with ID have had very few opportunities to participate in randomised trials about their healthcare, and no dedicated guidance has been published on undertaking a pilot randomised trial with this population. Therefore, our development of this protocol used general recommendations for clinical trial protocols proposed by Tetzlaff *et al*. [35] and the Consolidated Standards of Reporting Trials (CONSORT) 2010 guidelines [25]. This pilot randomised trial will enable us to:

1. Examine the feasibility of delivering the DESMOND-ID programme.
2. Assess eligibility, consenting rate, attendance levels, recruitment process, loss to follow-up, compliance and Hb1Ac of adults with ID and T2D.
3. Determine the acceptability of randomisation to adults with ID and their carers, using retention rates as a surrogate for acceptability [3].
4. Determine the appropriateness and the acceptability of the outcome measures to adults with ID through completion rates, in order to see whether the primary outcome measure (HbA1c) and secondary outcome measures (BMI, waist circumference, blood pressure, lipid profiles, CVD risk score, smoking, alcohol use, exercise, perceptions and severity of beliefs, and quality of life) can be collected.
5. Determine the appropriateness and acceptability of the outcome measures for the family and paid carers through completion rates.
6. Measure compliance of the educators in delivering the DESMOND-ID programme.
7. Estimate the treatment effect to determine whether this suggests a clinically important effect which will support the conduct of the full trial.

We will also conduct focus groups with adults with ID, their carers and educators to assess the acceptability of the intervention and the trial, and to explore their experience of the education programme, including any barriers and/or facilitators to adherence.

## Methods/Design

**Figure 1:**
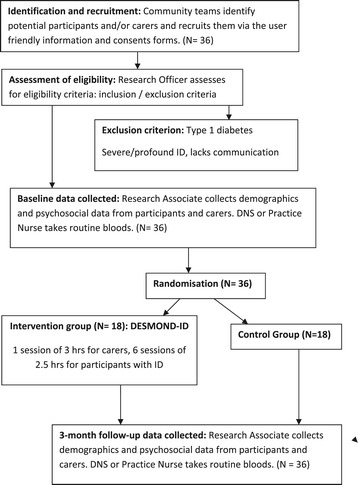
Flowchart of the study protocol. ID (intellectual disabilities).

The study is a two arm, individually randomised, pilot trial in adults with ID and T2D, and their family and/or paid carers. It compares the DESMOND-ID programme with usual routine care. Figure 1 illustrates the pathway through the trial. This trial corresponds to phase 2 of the Medical Research Council’s [18,19] guidelines for complex interventions: assessing feasibility.

### Recruitment

#### Participants

ID is defined by significant limitations in both intellectual functioning and adaptive behaviour, which cover many everyday social and practical skills, and it originates before the age of 18 years [1]. Adults with mild to moderate ID with T2D from three countries in the United Kingdom (Northern Ireland, Scotland and Wales) will be recruited. A sample of 36 individuals in total will be included in this pilot study: 18 people with ID in the intervention group and the same number in the control group (N = 18). There will be 12 participants from each country; the participants will all come from one area per country. Where there are family and/or paid carers, they will also be recruited, and participants with ID who do not have a carer will also be included.

#### Recruitment strategy

In a study that examined the demographics, health and diabetes quality-of-care indicators of 186 people with ID in Northern Ireland, Taggart *et al*. [32] highlighted the difficulties in identifying and recruiting this population.

Therefore, potential participants will be recruited from ID statutory services and can also be self-referred or referred by their GP, practice nurse, DSN or community ID nurse, or by the family or paid carer in response to posters and flyers about the project. These will be placed in ID day centres, health centres and so forth.

#### Inclusion criteria

The eligibility criteria will require that participants must be 18 years of age or older, living in the community, with mild to moderate ID and T2D, identified in their clinical notes by the ID team, GP, practice nurse or DSN. The definition of a family or paid carer is someone who is either a family relative or residential member of staff who engages in the support of the person with ID. Verbal and/or written consent will be required from people with ID and from their carers before they enter the study.

#### Exclusion criteria

Participants with any one of the following criteria will not be eligible: type 1 diabetes, a severe or profound ID as assessed by the community ID team, inability to communicate and inability to give their verbal and/or written consent.

#### Consent

Participants with ID will be screened for eligibility by the primary healthcare team or community ID nurse, who will provide the potential participants with a user friendly information sheet and consent form. Participants with a family and/or paid carer will also be provided with an information sheet and consent form. It is only after consent has been obtained that the research team will contact the participant and their carer to arrange baseline data collection.

### Randomisation

Participants with ID and their carers will be assigned to one of two conditions via the use of a computerised random allocation system (the RALLOC module within Stata 13 (StataCorp LP, London, England)) with concealment to ensure that the allocation is not made before the participant has given their consent and joined the study. Details of each participant and their carer will be telephoned through to a research secretary at the Ulster University, who is not connected to the study. They will use the information supplied by the study statistician to inform the research team of the allocation for the participant and, where appropriate, their carer.

### Intervention

#### DESMOND-ID programme

The DESMOND-ID programme is an amended version of the original DESMOND Programme that offers structured education to support adults with T2D in self-managing their condition. The original DESMOND Programme is based upon non-ID participants with T2D attending a six-hour education programme in a locality near them (that is, hospitals, community centres, health centres and so forth), over one day or two half-days.

A detailed description of how the DESMOND-ID intervention was developed and piloted has been written for publication (Taggart, Truesdale, Stacey-Martin, Carey, Scott, Coates, *et al*., unpublished work), which used the Template for Intervention Description and Replication (TIDieR) checklist and guide [14]. The DESMOND-ID programme has an additional introductory education session that is held separately for the family and paid carers in order to support their understanding about T2D and how it is managed. Carers gain an understanding of how the DESMOND-ID programme works and their specific role in supporting the person with ID throughout the programme.

The DESMOND-ID programme will be delivered in a day-centre or health centre, over six weeks, with one session per week, each lasting approximately two and a half hours. The participant with ID and their carer, if appropriate, will be encouraged to attend the sessions together. The education sessions will be delivered by two trained educators who have been given two-days of training, encompassing the DESMOND core newly diagnosed and DESMOND-ID programme training. The educators can be health facilitators, community ID nurses, DSN or dieticians. Two educators will be trained in each of the three countries.

The educational intervention focuses on the concepts of self-management and empowerment. The DESMOND programme is based upon the following theories:

1. Self-regulation theory, which focuses on individuals’ illness representations as a key determinant of their behavioural and emotional responses to illness.
2. Social learning theory, which focuses on individuals’ perceptions of their ability to carry out behaviours and support behaviour change through developing a personal action plan.
3. Dual process theory, which is used to guide the educational process of addressing individuals’ current understanding of diabetes. This process is used to actively engage participants in the learning process.

The educational content of DESMOND-ID mirrors that of the DESMOND Programme. The way in which educators deliver this content is adapted to make it accessible for people with ID, and it covers the following: what diabetes is, food choices, monitoring, physical activity, risks and complications and a self-management plan.

Each of the education sessions is composed of two 30 to 45 minute education sections, with a break in the middle for refreshments. Previous work has shown that flexibility is required in delivery and timing of the education sessions in order to meet individuals’ concentration levels and learning needs.

#### Control group

Participants with ID and their carers who are randomly allocated to the control group will receive usual routine care. Discussions among the research and clinical teams have highlighted that adults with ID and T2D and their carers will not be offered any form of structured education.

Routine care normally includes health centre visits every three months in which the person will see their primary healthcare team. Participants’ diabetes control is reviewed within the primary healthcare team and education is focused on problem solving and safety issues. All those randomised to the control group will continue to receive usual routine care, and be asked to complete the data gathering instruments and allow appropriately qualified members of the research team to access to their clinical results at baseline and during the follow-up period. If the programme is found to be effective, participants in the control group will be offered the DESMOND-ID education programme by the trained educators at the end of the study.

#### Training of educators delivering the intervention

Six educators will be recruited and trained to deliver the DESMOND-ID education programme; two educators in each country. A two-day educator training programme will be provided by experienced DESMOND Programme trainers involved in adapting the original DESMOND Programme for adults with ID. Followed by one day of observation and mentorship of educators delivered in each of the sites, no training will be offered to the primary healthcare team in the control group and they will not be responsible for any intervention patients.

### Measures for participants with intellectual disabilities

This trial will use the same measures as used in previous studies that have examined the original DESMOND Programme to assess biomedical and psychosocial outcomes [10,11,16,23,28]. This will allow for a comparison of the effects in the non-ID and ID populations. The selection of these measures also adheres to the core outcome set (COS) guidelines identified by Williamson *et al*. [40], which will allow us to compare and contrast the effects of different interventions in ways that maximise power and minimise bias.

#### Demographic data

A profile of the participants with ID will be collected at the pre-intervention stage in order to describe factors such as age, gender, with whom they live, functioning, communication, physical and mental health status, duration of diabetes, treatment regimen and self-monitoring practices.

#### Biomedical data

Data on HbA1c, BMI, waist circumference, blood pressure, blood lipid levels (total, high-density lipoprotein, low-density lipoprotein and cholesterol), frequency of health centre attendance and missed appointments will be collected from the study participants two weeks before the education programme, and at the three-month follow-up period directly after the intervention (see Figure 1). Weight (kilograms), height (cm) and waist circumference (cm) will be measured with participants dressed in lightweight clothing, and they will be asked to remove outer garments such as coats and jackets, shoes and belts for all measurements. Blood pressure and blood will be taken by the practice nurse or DSN in the participants’ health centre. No extra blood tests will be required, because blood samples are taken routinely at their regular health centre check-ups by the practice nurse or DNS every three months. The clinical data will be collected at the baseline and at the end of the follow-up period (Figure 1). A *pro forma* will be developed to facilitate rapid documentation of the required data so that it can be promptly forwarded to the research team.

#### Psychosocial or behaviour measures

Participants with ID will complete the following measures at baseline and at three months after the intervention:

1. The Illness Perception Questionnaire-Revised [39] will examine participants’ understanding of diabetes (illness coherence score), perception of the duration of their illness (timeline score) and the perception of their ability to affect the course of their diabetes (personal responsibility score). This scale has not been validated for adults with ID.
2. The Diabetes Illness Representation Questionnaire [27] will examine participants’ perceptions about the seriousness and impact of diabetes. This scale has not been validated for adults with ID.
3. The Summary of Diabetes Self-Care Activities Questionnaire [36] will examine participants’ diet and smoking patterns. This scale has not been validated for adults with ID.
4. The International Physical Activity Questionnaire [4] will examine participants’ physical activity. This scale has been validated for adults with ID.

The research team may need to support the person with ID by reading the instructions and items aloud. As part of the revision of the original DESMOND Programme, Taggart, Truesdale, Bunting, Coates, Clarke, Scott, *et al*. unpublished work) amended these standardised questionnaires in terms of wording, layout and employing a more user friendly Likert response for the items (using smiling faces instead of a numerical scale). The scales psychometric properties will be tested to examine if these instruments are stable for this population. The statistician will be blinded as to which participants are in the intervention and control groups.

### Measures for family and paid carers

#### Demographic data

A profile of the family and paid carers will be collected once at baseline, and will include age, relationship to the person with ID and physical and mental health status.

#### Psychosocial data

A slightly modified version of the Self-Efficacy for Diabetes Scale [12] will be used for family and paid carers to measure their perceived ability to manage the diabetes regime of the person with ID. A slightly modified version of the Diabetes Family Responsibility Questionnaire [2] will be used to measure the level of responsibility assumed by family and paid carers in managing the diabetes regime of the persons with ID.

Although they were not used in earlier studies of the original trials of the DESMOND Programme, these two instruments will allow the research team to determine whether the intervention affects the family or paid carer’s knowledge, perceived self-efficacy and responsibility in supporting the person with ID to manage his or her T2D.

#### Feasibility

We will record numbers and proportions for recruitment, consent, attendance, loss to follow-up and compliance (number of sessions attended) on a weekly basis, along with basic costs.

#### Adherence to treatment protocol

Data on the educators’ adherence to the DESMOND-ID manual will be collected through observation by an independent observer, using a checklist based on the components of the intervention.

### Follow-up

Follow-up assessments of all biomedical and psychosocial measurements will be undertaken three months directly after the intervention has been delivered, with adults with ID and their carers, for both groups across the three countries. The biomedical measurements will be collected by either the DNS or practice nurse, and the psychosocial measurements will be collected by the research associates.

#### Quantitative analysis

The information collected will be collated and analysed. Interpretation will centre on reflection about potential improvements to the content of the curriculum and the processes involved in providing education sessions.

#### Focus groups

Following the three-month follow-up period and the collection of the associated data, the six adults with ID and T2D and their carers who received the intervention from each country will be invited to participate in a focus group. The focus groups will explore participants’ experiences of the DESMOND-ID programme, the barriers and enablers to compliance with the intervention and the acceptability of all elements of the trial. Focus groups will also be held with the educators. The focus groups will be semi-structured and will last 45 to 60 minutes.

#### Qualitative analysis

The transcripts will be subjected to a thematic content analysis. In order to ensure the truthfulness and consistency of the focus groups, a range of methods will be employed. Firstly, the verbatim recording the focus groups and transcribing of the digital files will ensure consistent and accurate accounts of what the participants say about their experiences. Secondly, the data will be subjected to a thematic content analysis. Thirdly, to authenticate the key themes and sub-themes, as identified by the researcher, members of the research team will be asked to examine a random selection of the transcripts. This systematic approach will further increase the robustness of the qualitative data, thereby enhancing the transfer value of the findings.

### Refining of materials

Results from the quantitative and qualitative stages of this study will be used to revise the DESMOND-ID programme further. Findings will be fed back to the Research Advisory Steering Group, and final adjustments will be made to DESMOND-ID before the full-scale randomised trial.

### Ethical considerations

The main ethical consideration that needs to be kept in mind when undertaking a study with adults with ID is capacity to consent. Capacity to decide whether to participate and consent in the trial is of the upmost importance, and this will be assessed through discussions with the participant and/or their carers. User friendly information sheets and consent forms have been developed to explain the purpose and nature of the trial, and what is involved in the education programme, if recruited to this arm. Any questions that the person and/or their carer have will be discussed and answered. To assess the person’s ability to understand what is involved with this trial, the person with ID will be asked to recall the different parts of the study. Informed consent will then be obtained; however, persons unable to understand what is involved and unable to provide their consent will not be included in the study.

### Ethical approval, research governance and trial sponsorship

Ethical approval has been given for this study from the Office of Research Ethics Northern Ireland (reference number: 14/NI/1104). The trial has been registered and its International Standard Randomised Controlled Trials Number (ISRCTN) is ISRCTN93185560. The Northern Ireland Hub for Trials Methodology Research will offer support and guidance on governance and good clinical practice. Ulster University is the sponsor of the study.

## Discussion

To our knowledge, this is the first intervention study addressing structured diabetes education for adults with ID which is simultaneously targeting family and paid carers. Currently, there are no structured education programmes available for adults with ID with T2D, nor for those with type 1 diabetes. The National Institute for Health and Care Excellence Diabetes Guidelines (21) emphasises the importance of being offered a place on a structured education programme within six months of being diagnosed with T2D, in order to better self-manage and avoid complications. The lack of planning and availability of appropriate structured education programmes and educational materials for adults with ID and T2D will lead to additional illness and worse health. There are significant benefits to be gained if adapting structured education programmes shown to be effective in the non-ID population are beneficial for people with ID and other cognitive impairments.

There are a number of conceptual models to show that people with ID can successfully develop new knowledge and skills, and therefore change their lifestyle behaviours [34,41]. Taggart *et al*. [31] highlighted the importance of addressing the person’s intrinsic motivation, to want to change their health behaviours and to engage in self-help, possibly by using the techniques of motivational interviewing rather than enforcing motivation from external sources. Other successful techniques include offering both group and one-to-one sessions with more time flexibility, based on repetition, greater use of kinesics learning and role-play scenarios, and using family and/or paid carers to support the person with ID to maintain the behaviour changes over time. Wilson and Goodman [41] reported that adults with mild to moderate ID and co-morbid physical health conditions (such as diabetes, arthritis or hypertension), could successfully participate in chronic disease self-management programmes if such programmes are modified. The programme also improved this population’s access to healthcare.

The United Nations Convention on the Rights of Persons with Disabilities [37] highlights the universal impetus for health promotion for people with ID, which highlights the highest attainable standards of health without discrimination. The Convention [37] further states that parties shall ‘provide persons with disabilities with the same range, quality and standard of free or affordable healthcare and programmes as provided to other persons, including population-based public health programmes’. However, people with ID continue to be excluded from many population-based public health programmes, despite the clearly documented benefits of health promotion.

People with ID have had very few opportunities to participate in randomised trials about their healthcare. This protocol is also novel because it will include adults with ID and, where appropriate, their carers, and this will promote self-determination, independence and better self-management strategies. The trial will have both national and international relevance, helping to influence how governments and service providers support adults with ID, and their carers in proactively managing that person’s T2D. The need for an evidence-based approach in this area is more urgent than ever, given the likely increase in older persons with ID who will develop T2D, and the growing financial burdens on limited health services.

### Trial status

Recruitment will begin in March 2015 and is scheduled to last for eight months.

## Abbreviations

ID: Intellectual disabilities
T2D: Type 2 diabetes
DSN: Diabetes Specialist Nurse
GP: General Practitioner
